# Impact of Optic Pathway Gliomas on Puberty and Growth in Neurofibromatosis Type 1: A 20-Year Experience From a Tertiary Center

**DOI:** 10.1177/08830738251341591

**Published:** 2025-05-27

**Authors:** Robyn Haysom, Amish Chinoy

**Affiliations:** 1 Faculty of Medicine, Biology & Health, 5292 University of Manchester, Manchester, UK; 2 Department of Paediatric Endocrinology, Royal Manchester Children's Hospital, Manchester, UK

**Keywords:** growth delay, neurofibromatosis type 1, optic nerve glioma, precocious puberty

## Abstract

Children with neurofibromatosis type 1 have an increased incidence of optic pathway gliomas and central precocious puberty. This study explores whether the presence and location of optic pathway gliomas is associated with the changes in height and pubertal onset that are seen in these children. Retrospective analysis was undertaken of 75 individuals with a diagnosis of both neurofibromatosis type 1 and optic pathway gliomas, known to a single quaternary neurofibromatosis type 1 center, over a 20-year period. Central precocious puberty was more likely with optic pathway gliomas, observed in 28% of the cohort, and was associated with either optic chiasm involvement (*P* = .046) or bilateral optic pathway gliomas (*P* < .001). This is presumably due to disruption in the hypothalamic-pituitary axis. Height standard deviation scores were not significantly different from the general population. Increased clinical monitoring of pubertal status is consequently required for children with neurofibromatosis type 1 and an optic pathway glioma.

Neurofibromatosis type 1 is an autosomal dominant condition affecting ∼1 in 2500 live births. It occurs because of a loss of function variant in the neurofibromin gene on chromosome 17q11.2.^1^ Fifty percent of neurofibromatosis type 1 cases are spontaneous mutations, and 50% are inherited germline varients.^2^ Individuals with neurofibromatosis type 1 have an increased risk of tumors, including peripheral nerve sheath tumors, gliomas, leukemia, phaeochromocytoma, and gastrointestinal stromal tumors.^3,4^ In children with neurofibromatosis type 1, optic pathway gliomas are the most common type of tumor: they are present in 15% to 20% of the population with neurofibromatosis type 1, and form part of the recently revised diagnostic criteria.^5,6^ Optic pathway gliomas can occur at older ages; however, the average age of optic pathway glioma diagnosis is 5 years of age, with most optic pathway gliomas being diagnosed by 7 years of age.^7,8^

Neurofibromatosis type 1 is also associated with an increased risk of endocrine disorders, in particular central precocious puberty and short stature.^9^ Central precocious puberty occurs when the hypothalamic-pituitary-gonadal axis is prematurely activated, causing a child to experience the physiological changes of pubertal development at an earlier age.^10,11^

Five percent of children with neurofibromatosis type 1 have short stature (defined as the individual's height being >2 standard deviations below the mean for their age and sex) prepuberty, but this increases to around 20% in the latter part of their puberty.^12^ Literature suggests that although children with neurofibromatosis type 1 have normal growth velocity prior to puberty, their relative height and height velocity then drops below the general population during puberty.^9^

There is some literature to suggest an association between optic pathway gliomas and endocrine disorders in neurofibromatosis type 1.^13^ A study of 116 children with optic pathway gliomas in Italy saw that almost 30% had endocrine disorders, including central precocious puberty, growth hormone deficiency, diencephalic syndrome and growth hormone hypersecretion.^13,14^

A likely explanation for the potential impact of optic pathway gliomas on endocrine function is optic pathway gliomas’ proximity to, or potential involvement of the hypothalamus and pituitary gland, causing interference with the hypothalamic-pituitary axis.^5^ The hypothalamic-pituitary axis is important for both growth and puberty. Growth hormone is produced by the pituitary gland, which has an impact on bone growth and subsequently height, via insulin-like growth factor 1. Secretion of growth hormone is stimulated by growth hormone–releasing hormone from the hypothalamus and inhibited by somatostatin from the hypothalamus. Luteinizing hormone and follicle-stimulating hormone are released from the pituitary gland in a pulsatile manner following stimulation by gonadotrophin-releasing hormone from the hypothalamus and drive secretion of sex hormones (estrogen or testosterone) from the gonads. The timing of pubertal onset is complex, with many stimulatory and inhibitory factors influencing the gonadotrophin-releasing hormone neurons in the hypothalamus.^15^ Although there is a theoretical basis to suggest a relationship between optic pathway gliomas (which may affect the hypothalamic-pituitary axis) and growth or puberty, so far only small studies have explored the association in children with neurofibromatosis type 1 who have optic pathway gliomas.^8,16–19^

This study aims to add to the current literature by observing the association between children with neurofibromatosis type 1 and optic pathway gliomas, and their pubertal timing and growth. It also explores whether any specific features of the optic pathway gliomas affect their risk of developing endocrine disorders such as central precocious puberty. Our working hypothesis was that the presence of optic pathway gliomas would be associated with shorter stature and central precocious puberty, particularly if affecting the hypothalamus or optic chiasm (which lies close to the pituitary), as this would disrupt the hypothalamic-pituitary axis.

## Methods

This study is a 20-year retrospective review of children with neurofibromatosis type 1 and optic pathway gliomas at Manchester University NHS Foundation Trust (a nationally commissioned quaternary center for the management of individuals with complex neurofibromatosis type 1 in the north of the United Kingdom).

The study population was selected by identifying individuals who had a diagnosis of neurofibromatosis type 1 (based on National Institute of Health consensus criteria for diagnosis of neurofibromatosis type 1) *and* the presence of an optic pathway glioma (based on contrast magnetic resonance imaging [MRI]) from individuals within the service database.^6,20^

We collected information on children's vertical height (at optic pathway glioma diagnosis and most recently), parental height (if available), biological sex, age, pubertal timing, neurofibromatosis type 1 inheritance, optic pathway glioma location, and treatment for optic pathway gliomas (if applicable). Early puberty was defined in female children as breast development commencing <8 years and menarche <10 years and in male children as testicular volumes of ≥4 mL at <9 years, and was confirmed by biochemical testing (either pubertal levels of basal luteinizing hormone >0.6 U/L) or by gonadotrophin-releasing hormone stimulation testing (stimulated peak luteinizing hormone >6 U/L). Delayed puberty was defined as an absence of breast development >13 years in female children, and testicular volumes <4 mL at >14 years in male children. Height measurements were converted into a standard deviation score (SDS) using KIDS International Growth Study (KIGS) Auxology.

Statistical analysis was undertaken using SPSS v29 with *P* < .05 deemed to represent statistical significance. For continuous variables (which were all normally distributed), 1 or 2 sample t-tests and Pearson correlation coefficients were used. For categorical variables, Fishers test was used. We also used multiple logistic regression to adjust for possible confounders in a multivariable model to assess the impact of different factors on outcome (ie, central precocious puberty).

Because this study involved retrospective anonymized data collection, ethical approval, institutional board approval, and consent were not required, as per the National Health Society Health Research Authority.^21^

## Results

### Demographics

Seventy-five patients were included in our study (41 female, 34 male). Overall, 47% of the cohort had inherited neurofibromatosis type 1 from a parent, 40% had a de novo mutation, and 14% had an unknown inheritance. The mean age of optic pathway glioma diagnosis was 6.0 years of age (range 0.4-15.7 years, standard deviation [SD] 3.8). Seventy-five percent of the cohort were diagnosed with an optic pathway glioma under the age of 8 years, and 98% by age of 10 years. At the point of their most recent height measurements within the pediatric service, the cohort's mean age was 12 years (range 3.3-18.8 years, SD 4.3). The mean duration of follow-up in this cohort was 6.3 years (with a range 0.76-17.1 years, SD 3.9).

Optic pathway gliomas were located as follows: 85% (n = 64) involved the optic nerve, 71% (n = 53) involved the optic chiasm, 7% (n = 5) involved within the hypothalamus, 11% (n = 8) involved the optic tract, 8% (n = 6) involved the optic radiations, and 69% (n = 40) were bilateral in nature.

Twenty-nine percent (n = 22) received chemotherapy, 5% (n = 4) received surgical resection, and none received radiotherapy. Of those receiving chemotherapy, 11 received vinblastine, 9 had carboplatin, 7 had vincristine, 4 had cisplatin, 3 had trametinib, 2 had cyclophosphamide, 2 had irinotecan, 2 had avastatin, 2 had bevacizumab, and 1 had nilotinib.

### Puberty

Fifteen individuals (20%) were excluded from pubertal analysis, as they were too young to comment on pubertal outcome unless already precocious. Of the remainder, 28% (n = 17) had central precocious puberty (9 male and 8 female). Another 3% (n = 2) had delayed puberty (1 male, 1 female).

The location of optic pathway glioma did not influence the incidence of central precocious puberty, except when affecting the chiasm or when bilateral involvement was noted (Table 1). 76% percent of the children with central precocious puberty had an optic pathway glioma with optic chiasm involvement, with this feature increasing a patient's chances of having central precocious puberty 3-fold (from 24% to 76%). All of those with central precocious puberty had bilateral optic pathway gliomas. However, not all children with bilateral optic pathway glioma had central precocious puberty. Neurofibromatosis type 1 inheritance and chemotherapy did not influence the rate of central precocious puberty when analyzed (Table 1).

**Table 1.** Incidence of Central Precocious Puberty Based on Location, Treatment, and Inheritance of Optic Pathway Glioma (N = 17).

	Incidence of CPP, n (%)	*P* value
Optic nerve involvement		
Yes	15 (88)	>.99
No	2 (12)	
Optic chiasm involvement		
Yes	13 (76)	.046
No	4 (24)	
Hypothalamic involvement		
Yes	3 (18)	.07
No	14 (82)	
Optic tract involvement		
Yes	2 (22)	.62
No	15 (88)	
Optic radiation involvement		
Yes	1 (6)	>.99
No	16 (94)	
Laterality		
Bilateral	17 (100)	<.001
Not bilateral	0 (0)	
Chemotherapy		
Yes	4 (24)	>.99
No	13 (76)	
Neurofibromatosis type 1 inheritance		
De novo	8 (47)	.74
Familial	6 (35)	
Unknown	3 (18)	

Abbreviation: CPP, central precocious puberty.

### Height SDS

**Figure 1.**
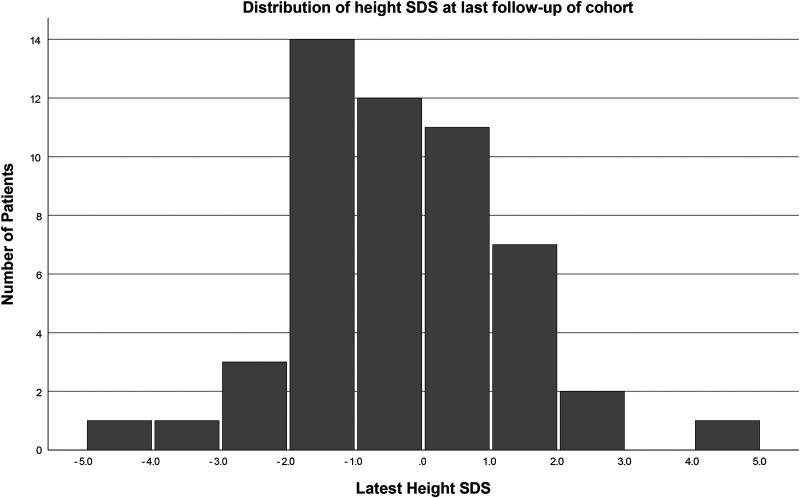
Distribution of height standard deviation score at last follow-up of cohort.

The mean height SDS of the cohort was −0.3 (SD 1.6, *P* = .28) at diagnosis and −0.4 (SD 1.4, *P* = .05) at the last follow-up (Figure 1). No difference in the latest height SDS is observed by demographics (Table 2).

**Table 2.** Differences in Height SDS at Latest Follow-up, by Different Demographics (Mode of Inheritance Sex, Age).

	Mode of Inheritance		Sex		Age
	Familial	De novo	Male	Female	
Mean height SDS	−0.7	−1.0	−0.2	−0.5	
Standard deviation	1.2	1.7	1.3	0.3	
Pearson correlation coefficient					−0.3
*P* value	.1		.39		0.8

Abbreviation: SDS, standard deviation score.

Central precocious puberty was found to be a direct factor that influenced height outcomes. We observed that the presence of central precocious puberty had a statistically significant influence on the height SDS of the cohort. Those with central precocious puberty had a mean height SDS +0.3 (SD 1.9), whereas those without central precocious puberty had a mean height SDS −0.7 (SD 1.3; mean difference −0.9, 95% confidence interval −1.8 to −0.04; *P* = .02).

The location or treatment of optic pathway gliomas generally did not seem to have an impact on height SDS, except a weak association with laterality whereby children with unilateral optic pathway gliomas were shorter than those with bilateral optic pathway gliomas (Table 3). However, this relationship between height SDS and laterality was due to the confounding factor of children with bilateral optic pathway gliomas being more likely to have central precocious puberty, which increased height SDS. When central precocious puberty is accounted for by multiple regression, no relationship is seen between optic pathway glioma laterality and height SDS (*r* = 0.16, *P* = .26).

**Table 3.** Differences in Latest Height SDS by Tumor Location and Treatment.

	Incidence, n	Mean height SDS	*P* value
Optic nerve			
Yes	56	−0.28	.93
No	9	−0.33	
Optic chiasm			
Yes	39	−0.67	.63
No	26	−0.04	
Hypothalamus			
Yes	5	−0.52	.7
No	60	−0.27	
Optic tract			
Yes	8	−0.25	.93
No	57	−0.30	
Optic radiations			
Yes	4	−0.82	.43
No	61	−0.25	
Laterality			
Unilateral	17	−0.88	.04
Bilateral	48	−0.08	
Chemotherapy			
Yes	20	−0.58 (0.33 SD)	.39
No	47	−0.25 (1.45 SD)	
Surgical resection			
Yes	4	−1.90	0.023
No	60	−0.21(1.40 SD)	

Abbreviations: SD, standard deviation; SDS, standard deviation score.

## Discussion

This retrospective study of 75 children shows that central precocious puberty occurs at much higher rates (28%) in children with both neurofibromatosis type 1 and optic pathway gliomas when compared to the general population or to children with neurofibromatosis type 1 without optic pathway gliomas. The rates of central precocious puberty were observed to be particularly significant in those with chiasmal or bilateral involvement. However, no impact on height SDS is noted, once the impacts of central precocious puberty is accounted for.

It is already well established that central precocious puberty occurs at a higher rate in the population with neurofibromatosis type 1 than in the general population. A Danish study in 2005 estimated central precocious puberty prevalence at 0.2% for girls and <0.05% for boys in the general population.^22,23^ On the other hand, Habiby et al observed in a cohort size of 219 that only 7 had central precocious puberty. Central precocious puberty, therefore, occurred in only 3% of their entire population with neurofibromatosis type 1; however, this was 39% of their population with neurofibromatosis type 1 and optic pathway gliomas (a potential underrepresentation of the true central precocious puberty figure given many of their cohort had not entered or completed puberty and so may have developed central precocious puberty in the future).^16^ Later, Virdis et al^24^ reviewed patients on the Italian neurofibromatosis type 1 register. They found the incidence of central precocious puberty in children without optic pathway gliomas matched that of the general population, but that children with neurofibromatosis type 1 with an optic pathway glioma had central precocious puberty at an incidence of 22%. With a neurofibromatosis type 1 cohort size of 412, 31 had optic pathway gliomas and only 7 had central precocious puberty. Neither study commented on the relationship between central precocious puberty and the tumor's exact locations. It is, however, readily agreed in the literature that central precocious puberty in children with neurofibromatosis type 1 is generally seen simultaneously with the presence of an optic pathway glioma. Furthermore, the few cases of central precocious puberty seen in the absence of an optic pathway glioma are potentially incidental or due to an evolving optic pathway glioma not yet evident on imaging from older literature that may have used older MRI scanning equipment. More recently, a study of 116 children with neurofibromatosis type 1 and optic pathway gliomas in Italy demonstrated a 20% rate of central precocious puberty, associated with hypothalamic involvement.^13^

Our cohort of individuals with neurofibromatosis type 1 (all with optic pathway gliomas), observed central precocious puberty at a rate of 28%. This incidence agrees with the association also observed in previous literature between optic pathway gliomas and central precocious puberty in children with neurofibromatosis type 1.^13,24^

We observed that if there was involvement of the optic chiasm or bilateral involvement in an individual's optic pathway glioma, this was a statistically significant predictor of potential central precocious puberty development. There was no statistically significant impact identified when optic pathway gliomas with hypothalamic involvement were isolated (*P* = .071). However, clinically 75% (3 of 4) of those with optic pathway gliomas within their hypothalamus had central precocious puberty, so this lack of significance may be secondary to our small sample size. This is in keeping with hypothalamic involvement demonstrated by Santoro et al.^13^ Only a few studies have previously explored the relationship between optic pathway glioma location and the development of central precocious puberty. Three of these studies found that all children with central precocious puberty and optic pathway gliomas had chiasmal involvement.^8,16,25^ The most recent of these looked more broadly at optic pathway gliomas in those with and without neurofibromatosis type 1. Gil Margolis et al^25^ observed central precocious puberty in 7.7% (n = 36) of their cohort of children with neurofibromatosis type 1 and 33.3% (n = 23) of their non–neurofibromatosis type 1 group. Across both cohorts in this study, all children with central precocious puberty had chiasmatic involvement.

There have previously been links made between central precocious puberty and chemotherapy, when Sani and Albanese^17^ observed 65% of children with neurofibromatosis type 1 who had had chemotherapy had general endocrinopathies compared with 36% who had not; however, it is difficult to know whether the difference between their results and ours is due to duration of follow-up, or the types of chemotherapy used.

The optic chiasm is located above the pituitary gland. Ordinarily, the hypothalamus-pituitary-axis will prevent the early onset of puberty. Therefore, we would hypothesize that an optic pathway glioma affecting the optic chiasm may interfere with this inhibition, thus causing puberty to commence early. Similarly, bilateral optic pathway gliomas may be more likely to interrupt these pathways compared with unilateral optic pathway gliomas.

It is also important to note that our cohort has a higher percentage of bilateral optic pathway gliomas (69%) compared with the reported rates of bilateral optic pathway gliomas in other populations with neurofibromatosis type 1 in the literature (34.8%).^26^ Although this does not discredit our findings of an association between bilateral optic pathway gliomas and central precocious puberty in children with neurofibromatosis type 1, it does have the potential to skew our data slightly, as the increased rate of bilateral optic pathway gliomas in our cohort may have contributed to our higher incidence of central precocious puberty.

Clinically, this result regarding central precocious puberty has implications in the management of children with neurofibromatosis type 1 following an optic pathway glioma diagnosis. Our study implies that children with neurofibromatosis type 1 who develop an optic pathway glioma have >25% chance of developing central precocious puberty. This is particularly the case for children with bilateral or chiasmal optic pathway gliomas, where the incidence of central precocious puberty is significantly greater. Thus, such children warrant much closer monitoring of their pubertal status during the prepubertal age ranges. Conversely, children with neurofibromatosis type 1 who develop central precocious puberty also carry a high likelihood of having an optic pathway glioma, and therefore an MRI scan of the brain becomes essential if an optic pathway glioma is not already diagnosed. Early diagnosis of central precocious puberty is important clinically so that treatment with gonadotrophin-releasing hormone partial agonists can be considered. This is to lessen the psychological burden for individuals and their families from early pubertal progress - for example, having an early onset of menstruation at an age when the child may not be emotionally mature enough to manage this. There is also data to support an impact on final adult height in children that develop CP <6 years.^27,28^ We were unable to determine whether those who were treated with gonadotrophin-releasing hormone analogs (n = 8) saw an effect on their final height versus those who did not, as the cohort was not followed up until final adult height.

Evidence of reduced height in populations with neurofibromatosis type 1 is noted in a North American study where 13% of the cohort had a height below −2 standard deviations.^29^ Further confirmation was observed by Zessis et al,^30^ whose patients had a significantly different mean height than the general population, accounted for by a reduced mean peak height velocity during puberty. Similarly, Clementi et al^31^ found height impairment in children with neurofibromatosis type 1 largely emerges after the age of 7 years in female children, and 12 years of age in male children. So, although the current literature finds individuals with neurofibromatosis type 1 statistically shorter than the general population, this difference is observed mainly during or after puberty.^30,31^ Given the average age for our height at diagnosis data was 6.0 years, most of the children had yet to enter puberty, hence they were not statistically short. The most recent height data were recorded at an average age of 12 years, and we observed that the heights at this point were on the border of being statistically short. It would therefore be productive to follow this cohort in a future study to observe whether their mean height SDS does indeed continue to drop off throughout or following puberty as the literature would suggest.

It is of note that in the majority of neurofibromatosis type 1 height studies, the cohorts contain children with and without optic pathway gliomas. Our study looked exclusively at children with both neurofibromatosis type 1 and optic pathway gliomas. It is important to recognize the higher incidence of central precocious puberty in this cohort may mask the expected short stature predicted from a population with neurofibromatosis type 1. Our data showed those with central precocious puberty were statistically taller than those without central precocious puberty, likely because of the earlier pubertal growth spurt in these children (which is likely to impair final height in the long term). On more specific analysis, laterality of the optic pathway glioma had a statistically significant influence on the latest height SDS of the cohort, with unilateral optic pathway gliomas causing children to be on average shorter, and bilateral optic pathway gliomas causing them to be taller. When regression analysis was conducted, it explained that this observation was indeed because of the confounding factor of central precocious puberty. Additionally, the high rates of central precocious puberty have likely also falsely increased the mean height SDS across the whole cohort and therefore may not provide a true reflection of the impact that optic pathway gliomas have on growth. Nonetheless, our overall data seem to suggest that optic pathway gliomas may not have as big an influence on height, and that the short stature in these individuals may simply be a part of their neurofibromatosis type 1 itself.

The main strength of our study lies in its relatively large sample size, with one of the largest cohorts of individuals with neurofibromatosis type 1 and optic pathway gliomas. There is some literature currently looking at growth and pubertal data specifically in children with neurofibromatosis type 1 and optic pathway gliomas, but little in those that have been longitudinally followed up. There are also scarce papers looking at predictive factors within optic pathway gliomas like tumor locations, gender, inheritance, or treatments. Therefore, this article provides meaningful additional data about the impact of optic pathway gliomas on height and, in particular, pubertal progress in a large cohort of children with neurofibromatosis type 1.

There are certain limitations of note in this study. Although the overall cohort size is relatively large, conducting sub-analysis with regard to optic pathway glioma locations and treatment did result in small numbers that affected interpretation. A larger study in the future, across centers, would mitigate this. Parental height is a considerable factor contributing to a child's final height. Only 14 individuals had parental heights on record, so all our analysis was done with height SDS without parental adjustment. Consequently, any height that can be attributed to parental tall or short stature is not accounted for and therefore there may be unidentified anomalies within the data set. This is particularly pertinent for those where one parent is also affected by neurofibromatosis type 1, and therefore may have additional reasons to be short, which may confound the data. There were certain variables that affect height that we did not fully account for, which may be relevant to children with neurofibromatosis type 1, such as limb length discrepancy and scoliosis. Finally, this study also relied on historical data given the original cohort spanned over 20 years. The UK population is trending toward being on average taller and entering puberty earlier,^32,33^ which we could not measure the impact of, or mitigate against, in this study.

## Conclusion

This 20-year retrospective study suggests that optic pathway gliomas in children with neurofibromatosis type 1 significantly increases the risk of central precocious puberty, particularly if chiasmatic or bilateral in nature. This is important for clinicians managing these individuals to be aware of so that they can appropriately monitor for this when children are in the prepubertal age range. No impact of optic pathway gliomas is suggested on height in children with neurofibromatosis type 1, implying that the short stature that is observed may be intrinsic to neurofibromatosis type 1 itself.
